# Exposure of U.S. Children to Residential Dust Lead, 1999–2004: II. The Contribution of Lead-Contaminated Dust to Children’s Blood Lead Levels

**DOI:** 10.1289/ehp.11918

**Published:** 2008-11-14

**Authors:** Sherry L. Dixon, Joanna M. Gaitens, David E. Jacobs, Warren Strauss, Jyothi Nagaraja, Tim Pivetz, Jonathan W. Wilson, Peter J. Ashley

**Affiliations:** 1 National Center for Healthy Housing, Columbia, Maryland, USA;; 2 Healthy Housing Solutions, Inc., Columbia, Maryland, USA;; 3 Battelle Memorial Institute, Columbus, Ohio, USA;; 4 U.S. Department of Housing and Urban Development, Washington, DC, USA

**Keywords:** blood lead, dust lead, housing, lead poisoning, National Health and Nutrition Examination Survey, NHANES

## Abstract

**Background:**

The U.S. Centers for Disease Control and Prevention collected health, housing, and environmental data in a single integrated national survey for the first time in the United States in 1999–2004.

**Objectives:**

We aimed to determine how floor dust lead (PbD) loadings and other housing factors influence childhood blood lead (PbB) levels and lead poisoning.

**Methods:**

We analyzed data from the 1999–2004 National Health and Nutrition Examination Survey (NHANES), including 2,155 children 12–60 months of age with PbB and PbD measurements. We used linear and logistic regression models to predict log-transformed PbB and the odds that PbB was ≥ 5 and ≥ 10 μg/dL at a range of floor PbD.

**Results:**

The population-weighted geometric mean (GM) PbB was 2.0 μg/dL (geometric standard error = 1.0). Age of child, race/ethnicity, serum cotinine concentration, poverty-to-income ratio, country of birth, year of building construction, floor PbD by floor surface and condition, windowsill PbD, presence of deteriorated paint, home-apartment type, smoking in the home, and recent renovation were significant predictors in either the linear model [the proportion of variability in the dependent variable accounted for by the model (*R*^2^) = 40%] or logistic model for 10 μg/dL (*R*^2^ = 5%). At floor PbD = 12 μg/ft^2^, the models predict that 4.6% of children living in homes constructed before 1978 have PbB ≥ 10 μg/dL, 27% have PbB ≥ 5 μg/dL, and the GM PbB is 3.9 μg/dL.

**Conclusions:**

Lowering the floor PbD standard below the current standard of 40 μg/ft^2^ would protect more children from elevated PbB.

The U.S. Department of Health and Human Services’ (DHHS) Healthy People 2010 initiative has set a national goal of eliminating blood lead (PbB) levels ≥ 10 μg/dL among children 1–5 years of age by 2010 (DHHS 2000). PbB used to define unsafe levels of exposure for children have decreased over the past few decades as additional evidence has demonstrated newly recognized adverse health effects, even at relatively low exposures [Canfield et al. 2003; Center for Disease Control and Prevention (CDC) 1991; Lanphear et al. 2005]. Childhood lead poisoning prevention efforts are sometimes called a victory in light of the dramatic reductions in population PbB. However, the magnitude of ongoing exposures, the remaining large stores of lead sources (particularly paint in older housing), and the length of time it has taken to address such exposures show that much remains to be done if a true, lasting victory is to be achieved (Jacobs et al. 2002; Lanphear 2007; Levin et al. 2008). We present new data on dust lead (PbD) loading and childhood PbB from the National Health and Nutrition Examination Survey (NHANES) 1999–2004 and examine their implications.

The most important source of lead exposure for children today is from lead paint as it deteriorates or is disturbed and subsequently contaminates settled residential dust and soil (Lanphear et al. 1998; Reissman et al. 2002). Another important source of lead in dust and soil is the estimated 5.9 million tons of gasoline lead emitted from motor vehicles before its removal in the mid-1980s (Mielke 1999). Normal hand-to-mouth activity exposes young children to lead in the residential environment (Bornschein et al. 1987; Lanphear et al. 1998). In 1999 and 2001, respectively, the U.S. Department of Housing and Urban Development (HUD) and the U.S. Environmental Protection Agency (EPA) established a PbD standard for the home environment of 40 μg/ft^2^, along with similar standards for windowsill PbD (250 μg/ft^2^) and lead in soil [400 parts per million (ppm) in play areas]. The previous guidance from U.S. EPA was 100 μg/ft^2^ for floor PbD (U.S. EPA 1995). Prior studies have firmly established the robust correlation of settled PbD on both floors and windowsills with children’s PbB (Davies et al. 1990; Lanphear et al. 1998; Wilson et al. 2007). However, analysis of exposure pathways shows that floor PbD has a direct effect on children’s PbB, with sill PbD having an indirect effect as mediated by floor PbD (HUD 2004). Until recently, nationally representative data for PbD and PbB (CDC 2005; Jacobs et al. 2002) were collected only in separate surveys. But between 1999 and 2004, NHANES interviewers collected PbD wipe samples and housing-related questionnaire data relevant to lead exposure from the homes of children 1–5 years of age. Blood samples from these children were collected at NHANES mobile examination centers and were analyzed for lead and other parameters. We examined the relationship between PbB in children and PbD on floors and window-sills and estimated PbB across the range of floor PbD in this nationally representative cross-sectional sample of children 1–5 years of age. This marks the first time that nationally representative data on environmental and biologic measurements for lead have been obtained in a single integrated survey. A companion article in this issue presents the predictors of residential PbD (Gaitens et al 2009).

## Methods

### Study population

We analyzed data from three waves of NHANES (1999–2000–2001–2002–2003–2004). NHANES is a nationally representative cross-sectional household survey that uses a complex, stratified, multistage probability sampling design to track the health of the noninstitutionalized civilian U.S. population. It has been a primary source of information about the national distribution of children’s PbB. Details of the NHANES protocol and all testing procedures are available elsewhere (NCHS 2006a, 2006b, 2006c). Our data set included 2,155 children 12 to 60 months of age with measured PbB. Only children living in housing built before 1978, when the United States banned the use of lead in residential paint, were included in the analysis of the influence of floor PbD on children’s PbB (*n =* 731).

### Child, household, and housing characteristics

NHANES interviewers collected data on age, race/ethnicity, sex, socioeconomic measures [family and household income and poverty-to-income ratio (PIR)], and other self-reported health data through a structured household interview. Participants self-reported their race and ethnicity. In this analysis, we used a composite race/ethnicity variable: non-Hispanic white, non-Hispanic black, Hispanic, or other race. These variables, as well as the housing characteristic variables, are described in the companion article. The PIR is the ratio of income to the family’s poverty threshold (Office of Management and Budget 1978). PIR values < 1.00 are below the poverty threshold, whereas PIR values of ≥ 1.00 indicate income above the poverty level. Variables on smoking behavior included the presence of smoking in the home, number of smokers, and the number of cigarettes smoked in the home per day. During their visit to the mobile examination center, NHANES participants provided venous blood samples, which were analyzed for PbB, serum cotinine, ferritin, iron, and total iron binding capacity.

NHANES measured PbB using graphite furnace atomic absorption spectrophotometry. The laboratory detection limit (DL) was 0.3 μg/dL. Only 0.23% of the sample results were below the DL. The DLs for cotinine were 0.05 ng/mL and 0.015 ng/mL for 1999–2000 and 2003–2004, respectively. For 2001–2002, there was a mixture of these two DLs. Twenty-six percent of the cotinine samples were below the DL. For all NHANES laboratory measurements, results below the DL were assigned the value of 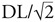.

### Statistical methods

Data were analyzed using SUDAAN (version 9.0.0; RTI International, Research Triangle Park, NC) and SAS (SAS System for Windows, version 9.1.3; SAS Institute Inc., Cary, NC). We used a linear regression model to predict natural log-transformed PbB and logistic regression models to predict the probability that a child’s PbB exceeded either 5 or 10 μg/dL. The models adjusted the parameter estimates for the clustering and unequal survey weights within NHANES. The modeling employed Taylor series expansion theory without degrees of freedom adjustments. Backward elimination of insignificant independent variables (*p* > 0.10) was followed by additional steps to allow addition and/or removal of variables. To provide an accurate prediction of children’s PbB without eliminating large fractions of the study sample because of missing values, we fit an intercept term for each variable that had a missing value. The overall *p*-value is the type 3 *F*-test that captures the overall statistical significance of each variable included in the model. For categorical variables with missing values, the missing level was not included in this hypothesis test.

Because NHANES collected serum cotinine only for children ≥ 3 years of age, many more children had questionnaire-based smoking data available than serum cotinine measurements. Therefore, we gave questionnaire-based smoking variables priority over measured serum cotinine levels.

Geometric mean (GM) PbB peaks between 18 and 36 months of age and slowly declines over the next few years, with the rate of decline varying in different populations (Dietrich et al. 2001; Tong et al. 1996; Wasserman et al. 1997). Based on the relationships between age and PbB observed in these studies, we determined that a quartic function of age of the child fit best.

Although most other analyses of the relationship between log PbB and log floor PbD were based on a linear relationship, the relationship may not be linear across the relatively low ranges observed in NHANES (Lanphear et al. 1998; U.S. EPA 1998, 2001). To investigate this further, we analyzed other data sets: the National Evaluation of the HUD Lead-Based Paint Hazard Control Grant Program (the Evaluation) (Galke et al. 2001; HUD 2004); the Rochester Lead-in-Dust Study (Rochester) (Lanphear et al. 1996a, 1996b); and the HUD National Risk Assessment Study (the RA Study) (Wilson et al. 2007). For each of these data sets and NHANES, we predicted log-PbB based on a cubic function of log floor PbD for children < 6 years of age (Table 1). The NHANES model accounted for clustering and unequal survey weights.

We predicted PbB at different PbD levels for children living in homes built before 1978 while controlling for other predictors of PbB using the aforementioned linear and logistic regression models and the population-weighted averages of covariates (except floor and sill PbD). For categorical variables, the levels were weighted according to their population-weighted relative frequency distribution. For continuous covariate variables with intercepts fit for missing values, the same percent of missing values observed in the population was assumed for the average risk values. For windowsill PbD values, we used a linear regression based on unweighted data from homes built before 1978 (*n =* 601). The correlation coefficient for the linear relationship between natural log-transformed sill and floor PbD is 0.38 (*p* < 0.001). The regression equation is: ln(sill PbD) = 2.654+0.524 × ln(floor PbD) (*r* = 0.38, mean square error = 2.78; SE for the intercept and slope are 0.070 and 0.053, respectively).

The GM PbB and the probability that PbB is ≥ 10 μg/dL and ≥ 5 μg/dL were predicted for floor PbD ranging from 0.25 to 40 μg/ft^2^ using the linear and logistic regression models, respectively. Although exponentiation of the predicted logarithm of the PbB may slightly overestimate the expected GM PbB, the large sample size minimizes the overestimation (Teekens and Koerts 1972).

## Results

### Characteristics of the study population

PbB data were available for 2,155 children 12–60 months of age. The population-weighted GM PbB was 2.03 μg/dL. Eight percent were ≥ 5 μg/dL, 1.71% were ≥ 10 μg/dL, and 0.33% were ≥ 15 μg/dL. Gaitens et al. (2009) present the descriptive statistics for PbD and additional housing variables. Here we present descriptive statistics for variables found to be significant (*p* < 0.10) in the PbB model (Tables 2 and 3). The weighted distribution shows that approximately 57% of the sampled population was non-Hispanic white, 15% was non-Hispanic black, and 24% was Hispanic. The vast majority (97.43%) of the children were born in the United States. Fifty-eight percent lived in a single-family detached house, and almost one-quarter lived in an apartment. Fifty-two percent of the homes for which data on the year of construction were available were built before 1978. Approximately 6% of homes were built before 1950 and had evidence of deteriorated paint (i.e., peeling, flaking, or chipping paint) inside. Ten percent of children lived in pre-1978 homes where window, cabinet, or wall renovation was completed in the preceding 12 months.

### PbB modeling results

**Figure 1:**
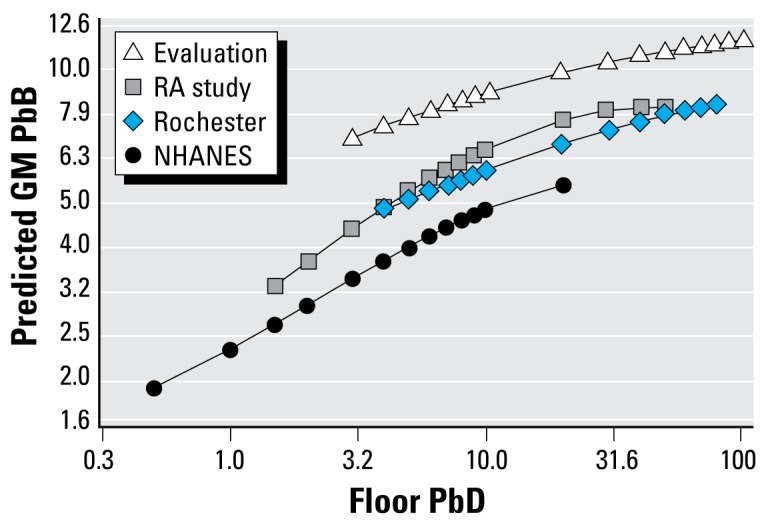
Predicted PbB (μg/dL) based on floor PbD (μg/ft^2^) by data set.

Although the models to predict log-PbB based on a cubic function of log floor PbD indicated that the cubic terms are not significant for two of the three data sets (the HUD Evaluation and Rochester), the quadratic terms were significant for all four data sets (Table 1). Figure 1 presents the predicted functions for the four data sets from the 5th to 95th floor PbD percentiles for each study except NHANES, which goes up to the 99.5th percentile (24.2 μg/ft^2^). The figure shows that the slope and curvature of the relationship between log floor PbD and log PbB observed for the NHANES data are similar to other studies.

Children’s PbB is strongly predicted by floor PbD and surface type and condition of floor (Table 4), with higher PbB associated with uncarpeted floors that were not smooth and not cleanable. Differences in the effect of PbD on PbB for uncarpeted smooth and cleanable, low-pile carpet and high-pile carpet were not significant, so these surfaces/conditions were combined. Natural log-transformed windowsill PbD, PIR, and age were also significant predictors of PbB.

Non-Hispanic black children had significantly higher PbB than non-Hispanic whites (*p* < 0.001). Country of birth was also a significant predictor of PbB, with Mexican-born associated with higher PbB (*p* = 0.003). Children living in apartment buildings with ≥ 10 units were found to have lower PbB than children living in single-family detached or attached dwellings (*p* = 0.005 and *p* = 0.022, respectively). As expected, children living in newer housing have significantly lower PbB compared with children living in housing built before 1940 (*p* < 0.001). Children living in homes built before 1978 that had renovation (within the preceding 12 months), which often disturbs paint lead, had higher PbB (*p* = 0.045).

Children who resided in a home where smoking occurred inside had significantly higher PbB than children who lived in homes with no smoking (*p* = 0.015). Even after controlling for the presence of smoking in the linear model, increasing log cotinine concentrations were associated with increasing PbB (*p* = 0.002).

Table 5 presents the logistic regression results for predicting PbB ≥ 5 μg/dL and ≥ 10 μg/dL. If a variable was significant in one logistic regression model but not the other model, the cells for the variable contain a dash (—). Although most of the variables that were significant in the linear regression model were also significant in the 5 μg/ dL logistic regression model, the 10 μg/dL logistic regression model identified fewer significant predictors. The proportion of variability in the dependent variable accounted for by the model (*R*^2^) for the 5 μg/dL and 10 μg/dL logistic models were much lower than for the linear model (16% and 5% vs. 40%, respectively). This result was attributable to the loss of information from using the dichotomous PbB outcomes in the logistic regression models and to the small number of children observed with PbB ≥ 10 μg/dL. The odds of having a PbB ≥ 5 μg/dL and ≥ 10 μg/ dL for non-Hispanic blacks were about twice those of non-Hispanic whites [odd ratio (OR) = 2.04 and 2.01, respectively]. The odds of a PbB ≥ 5 μg/dL for children born in Mexico were 11.69 times those of children born in the United States. However, country of birth was not a significant factor in predicting PbB ≥ 10 μg/dL. The odds of having a PbB ≥ 5 μg/dL were more than three times higher for children living in pre-1950 housing with renovation than for children living in other homes (OR = 3.33). The odds of having a PbB ≥ 10 μg/dL were more than three times higher for children living in pre-1950 housing with deteriorated paint inside than for children living in other homes (OR = 3.53).

### Floor PbD thresholds

Table 6 presents the model predictions for average children living in a pre-1978 home for a range of floor PbD after controlling for the covariates described above. At a floor PbD of 6 μg/ft^2^, the models predict that 2.7% of children have PbB ≥ 10 μg/dL and 16.5% have PbB ≥ 5 μg/ dL, and that the GM PbB is 3.4 μg/dL. When floor PbD is 12 μg/ft^2^, the models predict that 4.6% of children have PbB ≥ 10 μg/dL and 26.8% have PbB ≥ 5 μg/dL, and that the GM PbB is 3.9 μg/dL. The upper bound of the 90% confidence interval (CI) for a prediction approximates the 95% upper bound for the prediction. For example, when floor PbD is 12 μg/ft^2^, the 90% CI for the probability that PbB is ≥ 10 μg/dL is between 2.7 and 7.9%. This means that we are approximately 95% confident that the probability that PbB ≥ 10 μg/dL is < 7.9%. The information presented assumes that floor PbD is equal to the specified value. If floor PbD is less than the specified value, the predicted GM PbB and probabilities would be lower than those in Table 6.

## Discussion

We found the GM PbB for children 12–60 months of age in the United States between 1999 and 2004 was 2 μg/dL and that 20 children per 1,000 had PbB ≥ 10 μg/dL. A prior study analyzing NHANES data collected 1994–1998 found that 63 children per 1,000 had PbB ≥ 10 μg/dL (Bernard and McGeehin 2003). Our findings show that 81 children per thousand had PbB ≥ 5 μg/dL. Although there is a clear and significant decline over time in childhood lead exposure demonstrated by these prevalence estimates from NHANES, there is still an unacceptable number of children who are poisoned each year.

Age, race/ethnicity, PIR, and year of construction of housing all significantly predicted PbB of children, which is consistent with other studies (CDC 2005; Pirkle et al. 1994). Prior studies also found that PbB is typically higher in African-American children than in white children (Lanphear et al. 1996a, 2002; Raymond et al. 2007) and is higher in children living in poverty and in older homes (CDC 2005).

Previous studies using NHANES data have also documented the relationship between exposure to tobacco smoke and PbB (Bernard and McGeehin 2003; Mannino et al. 2003). Similar to our finding that serum cotinine was associated with PbB ≥ 10 μg/dL, Mannino et al. (2003) found that high levels of serum cotinine (a biomarker of exposure to environmental tobacco smoke) for older children 4–16 years of age was associated with PbB ≥ 10 μg/dL.

Prior studies have not demonstrated that children living in apartment buildings with ≥ 10 units are more likely to have lower PbB than children living in single-family detached houses. Although apartment buildings with ≥ 10 units tended to be of more recent construction than single-family detached homes and smaller apartment building (5%, 17%, and 78% constructed before 1940, respectively), not all the effect of home-apartment type is captured by the year of construction, because both variables are significant in the model. Although other studies suggest that lead hazards are more likely to be found in rental units than in owner-occupied properties (Jacobs et al. 2002), it is possible that owners of large apartment buildings may have more resources available for scheduled maintenance programs, which could help address lead hazards, compared with owners of smaller apartment buildings and single-family detached homes.

Despite having a relatively small number of children who were born outside the United States, our results indicate that Mexican-born was a strong predictor of PbB. A previous study examining the PbB of children living along the U.S.–Mexico border also found that children living in Mexico had higher PbB than children living in the United States. (Cowan et al. 2006). This finding may reflect continued use of lead-containing items imported from Mexico (e.g., pottery, foods, folk medicine) by families that recently resided there. Research has documented that use of these items can result in elevated PbB in children (CDC 1991).

Additionally, our study supports the association between PbB and renovation and floor and sill PbD, as expected. Other studies have shown that renovation activities can influence floor PbD (Reissman et al. 2002) and that floor PbD is a strong predictor of a child’s PbB (Davies et al. 1990; Lanphear et al. 1998; Rabinowitz et al. 1985; Wilson et al. 2007). The U.S. EPA (2008) recently promulgated a regulation intended to control lead exposures from renovation.

The rate of change in PbB with respect to floor PbD levels observed in this most recent NHANES analysis is similar to that found in three other studies analyzed here: the Evaluation, the RA Study, and the Rochester Study (Table 1). These other data sets are from higher-risk populations and therefore have higher PbD and PbB levels. The similarities in the PbB/PbD slope in the different studies indicate that it is reasonable to use the NHANES data to make inferences at higher floor PbD and PbB.

The current federal floor PbD standard of 40 μg/ft^2^ was established based on pre-1995 data from the Rochester Lead-in-Dust Study and a pooled analysis of 12 older epidemiologic studies using slightly different methods (HUD 1999; Lanphear et al. 1998; U.S. EPA 1998, 2001). The Rochester cohort and most of the studies comprising the pooled analysis were based on high-risk children and housing. The pooled analysis estimated that 95.3% of children 6–36 months of age would be protected from having a PbB ≥ 15 μg/ dL, using a floor PbD threshold of 40 μg/ ft^2^ and holding other sources of lead to their respective national averages in the residential environment (Lanphear et al. 1998). In the U.S. EPA analysis, the floor standard of 40 μg/ft^2^ was established jointly with standards for lead in windowsill dust, soil, and interior paint to protect at least 95% of children 12–30 months of age from developing a PbB ≥ 10 μg/dL when the windowsill and soil lead standards were also met (U.S. EPA 1998, 2001). Although the current 40 μg/ft^2^ standard was based on protecting children from developing high PbB (i.e., PbB ≥ 10 μg/dL or ≥ 15 μg/dL), the importance of preventing lower childhood lead exposure is illustrated by research that has demonstrated significant lead-related IQ decrements in children with PbB < 10 μg/dL (Canfield et al. 2003; Lanphear et al. 2005).

A strength of our study is that we were able to show the relationship of a range of floor PbD levels on children’s PbB while controlling for other significant predictors in a nationally representative sample of children. PbD and PbB from 1999 to 2004 were much lower than those observed in the earlier studies of higher-risk populations that were the foundation of the current floor PbD standard. In fact, these new data made the logistic model to predict PbB ≥ 10 μg/dL problematic, because only 2% of PbB (*n =* 51 of 2,155) were ≥ 10 μg/dL. Consequently, the percent of variation (*R*^2^) explained by the predictors in the 10 μg/dL logistic model was much lower than that of the linear model (*R*^2^ = 5% vs. *R*^2^ = 40%). We present the logistic regression model for 5 μg/dL because no other PbB thresholds have regulatory significance, and 11% of children had PbB ≥ 5 μg/dL (237 of 2,155 children; *R*^2^ = 16%). Iqbal et al. (2008) suggest that the threshold for elevated PbB may be lowered from 10 to 5 μg/dL and examines the impact of this reduction.

NHANES collected both health and environmental data from a nationally representative sample of children between 12 and 60 months of age; however, the NHANES data are not necessarily representative of the U.S. housing stock. Iqbal and collaborators (2008) found that for NHANES 1999–2002, a large number of children 1–5 years of age in NHANES (16.3%) had missing PbB values. Non-Hispanic white children, homeowners, and children from households with high income levels and with health insurance had a higher percentage of missing PbB values. This may have inflated the estimates of GM PbB and overestimated the prevalence of PbB ≥ 5 μg/dL and PbB ≥ 10 μg/dL.

In addition, NHANES collected only a single floor PbD measurement in each house. Although the single measurement was from the room in which the children spent the most time, the average of several floor dust samples would likely provide a more precise estimate of a child’s total exposure.

In this article we examined PbB across a range of floor PbD. An analysis of exposure pathways found that floor PbD has a direct effect on children’s PbB, whereas sill PbD has an indirect effect on children’s PbB as mediated by floor PbD (HUD 2004). In the NHANES data analyzed in this article, floor PbD is more predictive of PbB than sill PbD (*R*^2^ = 19.4% for floors; *R*^2^ = 11.9% for sills; *R*^2^ = 23.0% for floors and sills combined). When floor PbD = 12 μg/ft^2^, we show that 4.6% of children have PbB ≥ 10 μg/dL (Table 6). Based on the logistic model for 10 μg/dL, when floor PbD = 12 μg/ft^2^, sill PbD = 90 μg/ft^2^, and other covariates are at their national averages, the model predicts that 95% of children have PbB < 10 μg/dL. If homes have floor PbD below 12 μg/ft^2^ and sill PbD below 90 μg/ft^2^, less than 5% of children would have PbB ≥ 10 μg/dL.

The national estimate of the GM floor PbD in U.S. housing for 1998–2000 was 1.1 μg/ft^2^ (Jacobs et al. 2002). Furthermore, data from high-risk houses in the HUD evaluation study showed that PbD on floors continued to decline after the intervention, dropping from a GM of 14 μg/ft^2^ immediately after intervention to a GM of only 4.8 μg/ft^2^ 6 years after hazard control (Wilson et al. 2006). Together, these data demonstrate that floor PbD is well below the current federal standard of ≤ 40 μg/ft^2^ for the vast majority of houses.

Historically, allowable PbD levels have declined as research has progressed. In the early 1990s, Maryland enacted a floor PbD standard of ≤ 200 μg/ft^2^ (Code of Maryland 1988). U.S. EPA issued guidance in 1995 lowering the floor PbD level to ≤ 100 μg/ft^2^, and in 1999–2001, HUD and U.S. EPA promulgated a floor PbD standard of ≤ 40 μg/ft^2^, which has remained unchanged. Our findings suggest that floor and windowsill PbD should be kept as low as possible. Levels of PbD on floors between 6 μg/ft^2^ and 12 μg/ft^2^ can be expected to protect most children living in pre-1978 homes from having PbB ≥ 10 μg/dL. Protection at lower PbB would require lower PbD.

## Correction

The values given in the first sentence of “Results” were incorrect in the original manuscript published online. They have been corrected here.

## Figures and Tables

**Table 1:** Models predicting children’s log PbB based on floor PbD.

		Data set			
Statistic	Term	Evaluation^a^	NHANES	RA Study^b^	Rochester^c^
Regression	Intercept	1.664 (0.073)	0.826 (0.023)	0.938 (0.193)	1.168 (0.194)
Coefficient (SE)	Log (floor PbD)	0.269 (0.042)	0.319 (0.029)	0.491 (0.293)	0.340 (0.103)
		*p* < 0.001	*p* < 0.001	*p* = 0.096	*p* = 0.003
	[Log (floor PbD)]^2^	−0.022 (0.006)	0.033 (0.008)	0.003 (0.117)	−0.021 (0.012)
		*p* = 0.001	*p* < 0.001	*p* = 0.980	*p* = 0.083
	[Log (floor PbD)]^3^	—	−0.014 (0.004)	−0.009 (0.013)	—
			*p* < 0.001	*p* = 0.498	
Overall *p*-value for log (floor PbD)		*p* < 0.001	*p* < 0.001	*p* < 0.001	*p* < 0.001
*R*^2^		6.9	23.6	23.3	8.6
Mean-square error		0.512	0.262	0.532	0.350
No. of children/units		1,096	2,065	203	205

a Data from Galke et al. (2001), U.S. HUD (2004).

b Data from Wilson et al. (2007).

c Data from Lanphear et al. (1996a, 1996b).

**Table 2:** Descriptive statistics for PbB, housing, and demographic variables, NHANES 1999–2004.

		All homes			Pre-1978 homes		
			Weighted			Weighted	
Variable	Level	No.	GM (GSE)	AM (SE)	No.	GM (GSE)	AM (SE)
PbB (μg/dL)	—	2,155	2.03 (1.03)	2.51 (0.09)	731	2.16 (1.03)	2.69 (0.10)
Age (months)	—	2,155	33.6 (1.01)	36.7 (0.35)	731	33.4 (1.02)	36.6 (0.64)
Cotinine (ng/mL)	Missing	1,326	—	—	449	—	—
	Nonmissing	829	0.18 (1.14)	1.02 (0.11)	282	0.18 (1.18)	0.97 (0.20)
Floor surface/condition^a^ × floor PbD (μg/ft^2^ )	Missing	90	—	—	0	—	—
	Not smooth and cleanable	25	1.70 (1.47)	4.92 (2.11)	8	1.26 (1.69)	4.67 (3.60)
	Smooth and cleanable or carpeted	2,040	0.52 (1.05)	1.34 (0.14)	723	0.64 (1.07)	1.78 (0.31)
	All nonmissing	2,065	0.52 (1.05)	1.34 (0.14)	731	0.64 (1.07)	1.80 (0.31)
PIR^b^	Missing	136	—	—	24	—	—
	Nonmissing	2,019	—	2.07 (0.05)	707	—	2.25 (0.09)
Windowsill PbD (μg/ft^2^ )	Missing	537	—	—	130	—	—
	Nonmissing	1,618	7.64 (1.07)	57.8 (9.42)	601	10.5 (1.11)	71.8 (14.8)

Abbreviations: AM, arithmetic mean; GSE, geometric standard error.

a Table 1 in the companion article presents descriptive statistics by the expanded groups of floor surface/condition.

b GM and GSE are undefined because of zero values.

**Table 3:** Descriptive statistics for PbB, housing, and demographic categorical variables, NHANES 1999–2004.

		All homes			Pre-1978 homes		
			Weighted percent			Weighted percent	
Variable	Levels	No.	Missing included	Missing excluded	No.	Missing included	Missing excluded
PbB ≥ 5 μg/dL	No	1,918	91.88	91.88	643	90.84	90.84
	Yes	237	8.12	8.12	88	9.16	9.16

PbB ≥ 10 μg/dL	No	2,104	98.29	98.29	708	97.97	97.97
	Yes	51	1.71	1.71	23	2.03	2.03

PbB ≥ 15 μg/dL	No	2,140	99.67	99.67	725	99.65	99.65
	Yes	15	0.33	0.33	6	0.35	0.35

Home-apartment type	Missing	39	1.77	—	7	0.47	—
	Mobile home or trailer	205	9.77	9.95	20	2.69	2.70
	One-family house, detached	1,047	57.19	58.23	490	72.93	73.27
	One-family house, attached	218	9.21	9.38	82	9.93	9.98
	Apartment (1–9 units)	302	10.40	10.59	60	6.98	7.01
	Apartment (≥ 10 units)	344	11.65	11.86	72	7.00	7.03

Year of construction	Missing	840	28.10	—	—	—	—
	1990–present	287	19.61	27.28	—	—	—
	1978–1989	265	14.84	20.64	—	—	—
	1960–1977	304	14.35	19.96	300	39.43	39.43
	1950–1959	168	7.43	10.34	158	19.38	19.38
	1940–1949	82	4.27	5.94	76	11.00	11.00
	Before 1940	209	11.39	15.84	197	30.19	30.19

Anyone smoke inside the home	Missing	23	1.50	—	1	0.46	—
	Yes	430	20.78	21.09	159	22.59	22.69
	No	1,702	77.73	78.91	571	76.95	77.31

Presence of deteriorated paint inside pre-1950 home^a^	Missing	239	7.87	—	0	—	—
	Yes	121	5.99	6.50	112	15.64	15.64
	No	1,795	86.14	93.50	619	84.36	84.36

Window, cabinet, or wall renovation in pre-1978 home^b^	Missing	176	6.02	—	9	0.64	—
	Yes	175	9.72	10.34	166	26.34	26.51
	No	1,804	84.26	89.66	556	73.02	73.49

Window, cabinet, or wall renovation in pre-1950 home^c^	Missing	174	5.97	—	7	0.49	—
	Yes	70	3.98	4.23	65	10.69	10.74
	No	1,911	90.05	95.77	659	88.82	89.26

Race/ethnicity	Non-Hispanic white	618	57.09	57.09	252	64.14	64.14
	Non-Hispanic black	634	15.32	15.32	188	12.54	12.54
	Hispanic^d^	837	23.82	23.82	265	20.01	20.01
	Other	66	3.77	3.77	26	3.31	3.31

Country of birth	Missing	4	0.19	—	1	0.09	—
	United States	2,088	97.25	97.43	715	98.28	98.38
	Mexico	39	0.87	0.87	7	0.43	0.43
	Elsewhere	24	1.70	1.70	8	1.19	1.19

a Yes = presence of deteriorated paint inside AND pre-1950 home; no = no deteriorated paint inside OR post-1950 home.

b Yes = window, cabinet, or wall renovation AND pre-1978 home; no = no renovation OR post-1978.

c Yes = window, cabinet, or wall renovation AND pre-1950 home; no = no renovation OR post-1950.

d Sixty-six percent of Hispanics are Mexican Americans.

**Table 4:** Linear model results for log children’s PbB.^a^

Variables	Overall *p*-value	Levels	Estimate (SE)	*p*-Value
Intercept	0.172		−0.517 (0.373)	0.172
Age (in years)	< 0.001	Age	2.620 (0.628)	< 0.001
		Age^2^	−1.353 (0.354)	< 0.001
		Age^3^	0.273 (0.083)	0.002
		Age^4^	−0.019 (0.007)	0.008
Year of construction	0.014	Intercept for missing	−0.121 (0.052)	0.024
		1990–present	−0.198 (0.058)	0.001
		1978–1989	−0.196 (0.060)	0.002
		1960–1977	−0.174 (0.056)	0.003
		1950–1959	−0.207 (0.065)	0.003
		1940–1949	−0.012 (0.072)	0.870
		Before 1940	0.000	—
PIR	< 0.001	Intercept for missing	0.053 (0.065)	0.420
		Slope	−0.053 (0.012)	< 0.001
Race/ethnicity	< 0.001	Non-Hispanic white	0.000	—
		Non-Hispanic black	0.247 (0.035)	< 0.001
		Hispanic	−0.035 (0.030)	0.251
		Other	0.128 (0.070)	0.073
Country of birth	0.002	Missing	−0.077 (0.219)	0.728
		United States^b^	0.000	—
		Mexico	0.353 (0.097)	< 0.001
		Elsewhere	0.154 (0.121)	0.209
Floor surface/condition × log floor PbD	< 0.001	Intercept for missing	0.178 (0.094)	0.065
		Not smooth and cleanable	0.386 (0.089)	< 0.001
		Smooth and cleanable or carpeted	0.205 (0.032)	< 0.001
Floor surface/condition × (log floor PbD)^2^		Not smooth and cleanable	0.023 (0.015)	0.124
		Smooth and cleanable or carpeted	0.027 (0.008)	0.001
Floor surface/condition × (log floor PbD)^3^		Uncarpeted not smooth and cleanable	−0.020 (0.014)	0.159
		Smooth and cleanable or carpeted	−0.009 (0.004)	0.012
Log windowsill PbD	0.002	Intercept for missing	0.053 (0.040)	0.186
		Slope	0.041 (0.011)	< 0.001
Home-apartment type	< 0.001	Intercept for missing	−0.064 (0.097)	0.511
		Mobile home or trailer	0.127 (0.067)	0.066
		One family house, detached	−0.025 (0.046)	0.596
		One family house, attached	0.000	—
		Apartment (1–9 units)	0.069 (0.060)	0.256
		Apartment (≥ 10 units)	−0.133 (0.056)	0.022
Anyone smoke inside the home	0.015	Missing	0.138 (0.140)	0.331
		Yes	0.100 (0.040)	0.015
		No	0.000	—
Log cotinine concentration (ng/dL)	0.004	Intercept for missing	−0.150 (0.063)	0.023
		Slope	0.039 (0.012)	0.002
Window, cabinet, or wall renovation in a pre-1978 home	0.045	Missing	−0.008 (0.061)	0.896
		Yes	0.097 (0.047)	0.045
		No	0.000	—

a *n* = 2,155; *R*^2^ = 40%.

b Includes the 50 states and the District of Columbia.

**Table 5:** Model results for log odds children’s PbB ≥ 5 μg/dL and ≥ 10 μg/dL.^a^

		PbB ≥ 5 μg/dL			PbB ≥ 10 μg/dL		
Term	Levels	Overall *p*-value	Estimate (SE)	*p*-Value	Overall *p*-value	Estimate (SE)	*p*-Value
Intercept		0.005	−13.004 (4.365)	0.005	0.048	−14.170 (6.976)	0.048
Age (in months)	Age	0.007	18.783 (7.069)	0.011	0.068	14.703 (11.140)	0.194
	Age^2^		−10.455 (4.039)	0.013		−6.801 (6.673)	0.314
	Age^3^		2.358 (0.959)	0.018		1.170 (1.687)	0.492
	Age^4^		−0.189 (0.081)	0.024		−0.066 (0.149)	0.659
PIR	Intercept for missing	0.006	0.319 (0.444)	0.477	—	—	—
	Slope		−0.267 (0.099)	0.010		—	—
Race/ethnicity	Non-Hispanic white	0.003	0.000		0.038	0.000	
	Non-Hispanic black		0.712 (0.303)	0.023		0.696 (0.373)	0.068
	Hispanic		−0.468 (0.336)	0.171		−0.590 (0.513)	0.257
	Other		−0.048 (0.928)	0.959		−0.118 (1.002)	0.907
Country of birth	Intercept for missing	0.002	−0.518 (1.140)	0.652	—	—	—
	United States^b^		0.000	—	—	—	—
	Mexico		2.459 (0.641)	< 0.001	—	—	—
	Elsewhere		0.113 (1.145)	0.922	—	—	—
Log floor PbD	Intercept for missing	< 0.001	0.989 (0.410)	0.020	< 0.001	1.405 (0.630)	0.031
	Slope		0.807 (0.133)	< 0.001		0.710 (0.155)	< 0.001
Log windowsill PbD	Intercept for missing	0.056	0.466 (0.336)	0.172	0.071	1.234 (0.653)	0.066
	Slope		0.198 (0.080)	0.017		0.242 (0.102)	0.022
Home-apartment type	Intercept for missing	0.029	−0.434 (0.727)	0.553	0.048	1.638 (0.802)	0.047
	Mobile home or trailer		−0.078 (0.428)	0.857		0.480 (0.605)	0.432
	One-family house, detached		−0.373 (0.295)	0.214		0.212 (0.357)	0.556
	One-family house, attached		0.000	—		0.000	—
	Apartment (1–9 units)		−0.276 (0.361)	0.449		0.334 (0.508)	0.515
	Apartment (≥ 10 units)		−1.022 (0.326)	0.003		−1.173 (0.569)	0.045
Window, cabinet, or wall renovation in pre-1950 home	Missing	0.004	−0.052 (0.320)	0.872	—	—	—
	Yes		1.203 (0.399)	0.004	—	—	
	No		0.000	—		—	—
Presence of deteriorated paint inside a pre-1950 home	Intercept for missing	—	—	—	0.019	−0.012 (0.292)	0.968
	Yes		—	—	—	1.263 (0.520)	0.019
	No		—	—	—	0.000	—
Log cotinine concentration (ng/dL)	Intercept for missing	< 0.001	−0.299 (0.378)	0.434	0.006	−1.074 (0.931)	0.255
	Slope		0.483 (0.117)	< 0.001		0.455 (0.153)	0.005

a *n* = 2,155; *R*^2^ = 16% and 5%. Approximate *R*^2^ from Cox–Snell methodology.

b Includes the 50 states and the District of Columbia.

**Table 6:** Estimated PbB for children living in pre-1978 housing by floor PbD, NHANES 1999–2004.

Floor PbD (μg/ft^2^ )	Percent of homes ≥ floor PbD	GM PbB^a^	Probability (%)PbB ≥ 10 μg/dL^b^	Probability (%)PbB ≥ 5 μg/dL^c^
0.25	79.1	1.7 (1.6–1.8)	0.2 (0.1–0.6)	1.1 (0.7–1.8)
0.50	55.4	1.9 (1.8–2.0)	0.4 (0.1–1.0)	2.1 (1.4–3.1)
1.00	30.5	2.2 (2.1–2.3)	0.6 (0.3–1.5)	3.8 (2.7–5.5)
1.50	21.8	2.4 (2.3–2.6)	0.9 (0.4–1.9)	5.4 (3.7–7.9)
2	16.7	2.6 (2.4–2.8)	1.1 (0.6–2.2)	6.9 (4.6–10.2)
4	8.0	3.1 (2.8–3.4)	2.0 (1.1–3.5)	12.1 (7.7–18.5)
5	4.9	3.3 (2.9–3.6)	2.3 (1.3–4.1)	14.4 (9.0–22.2)
6	4.2	3.4 (3.0–3.8)	2.7 (1.5–4.7)	16.5 (10.2–25.6)
7	3.7	3.5 (3.1–4.0)	3.0 (1.7–5.3)	18.5 (11.3–28.7)
8	3.5	3.6 (3.2–4.1)	3.4 (2.0–5.8)	20.3 (12.3–31.7)
9	3.3	3.7 (3.3–4.2)	3.7 (2.1–6.4)	22.1 (13.3–34.4)
10	3.0	3.8 (3.3–4.3)	4.0 (2.3–6.9)	23.8 (14.2–36.9)
12	2.5	3.9 (3.4–4.5)	4.6 (2.7–7.9)	26.8 (16.0–41.5)
14	2.1	4.0 (3.5–4.7)	5.2 (3.0–9.0)	29.6 (17.5–45.5)
16	1.4	4.1 (3.6–4.8)	5.8 (3.3–10.0)	32.2 (19.0–49.0)
18	1.3	4.2 (3.6–4.9)	6.4 (3.6–11.0)	34.5 (20.3–52.1)
20	1.3	4.3 (3.6–5.0)	6.9 (3.9–11.9)	36.6 (21.6–54.9)
22	1.2	4.3 (3.7–5.1)	7.4 (4.1–12.9)	38.6 (22.8–57.3)
24	1.2	4.4 (3.7–5.2)	7.9 (4.4–13.8)	40.5 (23.9–59.6)
26	0.7	4.4 (3.7–5.2)	8.4 (4.6–14.8)	42.2 (25.0–61.6)
28	0.7	4.4 (3.7–5.3)	8.9 (4.8–15.7)	43.8 (26.0–63.5)
30	0.7	4.4 (3.7–5.4)	9.3 (5.1–16.6)	45.4 (26.9–65.2)
32	0.6	4.5 (3.7–5.4)	9.8 (5.3–17.4)	46.8 (27.9–66.7)
34	0.6	4.5 (3.7–5.5)	10.2 (5.5–18.3)	48.1 (28.7–68.1)
36	0.5	4.5 (3.6–5.6)	10.7 (5.7–19.1)	49.4 (29.6–69.4)
38	0.4	4.5 (3.6–5.6)	11.1 (5.9–20.0)	50.6 (30.4–70.6)
40	0.4	4.5 (3.6–5.7)	11.5 (6.1–20.8)	51.8 (31.2–71.8)

a 90% CI based on the linear model for log PbB.

b 90% CI based on the logistic model for PbB ≥ 10 μg/dL.

c 90% CI based on the logistic model for PbB ≥ 5 μg/dL.

## References

[b1-ehp-117-468] Bernard SM, McGeehin MA (2003). Prevalence of blood lead levels ≥ 5 μg/dL among US children 1 to 5 years of age and socioeconomic and demographic factors associated with blood of lead levels 5 to 10 μg/dL, Third National Health and Nutrition Examination Survey, 1988–1994. Pediatrics.

[b2-ehp-117-468] Bornschein RL, Succop P, Kraft KM, Clark CS, Peace B, Hammond PB, Hemphill DD (1987). Exterior surface lead, interior house dust lead and childhood exposure in an urban environment. Trace Substances in Environmental Health.

[b3-ehp-117-468] Canfield RL, Henderson CR, Cory-Slechta DA, Cox C, Jusko TA, Lanphear BP (2003). Intellectual impairment in children with blood lead concentrations below 10 microg per deci-liter. N Engl J Med.

[b4-ehp-117-468] CDC (1991). Preventing Lead Poisoning in Young Children: A Statement by the Centers for Disease Control.

[b5-ehp-117-468] CDC (Centers for Disease Control and Prevention) (2005). Blood lead levels – United States, 1999–2002. MMWR Morb Mortal Wkly Rep.

[b6-ehp-117-468] Code of Maryland (1988). Procedures for Abating Lead Containing Substances from Buildings.

[b7-ehp-117-468] Cowan L, Esteban E, McElroy-Hart R, Kieszak S, Meyer PA, Rosales C (2006). Binational study of pediatric blood lead levels along the United States/Mexico border. Int J Hyg Environ Health.

[b8-ehp-117-468] Davies D, Thornton I, Watt J, Culbard E, Harvey P, Delves H (1990). Lead intake and blood lead in two-year-old UK urban children. Sci Total Environ.

[b9-ehp-117-468] DHHS (U.S. Department of Health and Human Services) (2000). Healthy People 2010. With Understanding and Improving Health and Objectives for Improving Health 2 vols.

[b10-ehp-117-468] Dietrich JN, Ris MD, Succop PA, Berger OG, Bornschein RL (2001). Early exposure to lead and juvenile delinquency. Neurotoxicol Teratol.

[b11-ehp-117-468] Gaitens JM, Dixon SL, Jacobs DE, Nagaraja J, Strauss W, Wilson JW (2009). Exposure of U.S. children to residential dust lead, 1999–2004: I. Housing and demographic factors. Environ Health Perspect.

[b12-ehp-117-468] Galke W, Clark CS, Wilson J, Jacobs D, Succop P, Dixon S (2001). Evaluation of the HUD Lead Hazard Control Grantee Program: early overall findings. Environ Res.

[b13-ehp-117-468] HUD (U.S. Department of Housing and Urban Development) (1999). Requirements for Notification, Evaluation and Reduction of Lead-Based Paint Hazards in Federally Owned Residential Property and Housing Receiving Federal Assistance. Final Rule. 24 CFR Part 35 Preamble. Fed Reg.

[b14-ehp-117-468] HUD (2004). Evaluation of the HUD Lead-Based Paint Hazard Control Grant Program: Final Report.

[b15-ehp-117-468] Iqbal S, Muntner P, Batuman V, Rabito FA (2008). Estimated burden of blood lead levels ≥ 5 μg/dl in 1999–2002 and declines from 1988 to 1994. Environ Res.

[b16-ehp-117-468] Jacobs DE, Clickner RP, Zhou JY, Viet SM, Marker DA, Rogers JW (2002). The prevalence of lead-based paint hazards in U.S. housing. Environ Health Perspect.

[b17-ehp-117-468] Lanphear BP (2007). The conquest of lead poisoning: a pyrrhic victory. Environ Health Perspect.

[b18-ehp-117-468] Lanphear BP, Hornung R, Ho M, Howard CR, Eberly S, Knauf K (2002). Environmental lead exposure during early childhood. J Pediatr.

[b19-ehp-117-468] Lanphear BP, Hornung R, Khoury J, Yolton K, Baghurst P, Bellinger DC (2005). Low-level environmental lead exposure and children’s intellectual function: an international pooled analysis. Environ Health Perspect.

[b20-ehp-117-468] Lanphear BP, Matte TD, Rogers J, Clickner RP, Dietz B, Bornschein RL (1998). The contribution of lead-contaminated house dust and residential soil to children’s blood lead levels. A pooled analysis of 12 epidemiologic studies. Environ Res.

[b21-ehp-117-468] Lanphear BP, Weitzman M, Eberly S (1996a). Racial differences in urban children’s environmental exposures to lead. Am J Public Health.

[b22-ehp-117-468] Lanphear BP, Weitzman M, Winter NL, Eberly S, Yakir B, Tanner M (1996b). Lead-contaminated house dust and urban children’s blood lead levels. Am J Public Health.

[b23-ehp-117-468] Levin R, Brown MJ, Kashtock ME, Jacobs DE, Whelan EA, Rodman J (2008). U.S. children’s lead exposures, 2008: implications for prevention. Environ Health Perspect.

[b24-ehp-117-468] Mannino DM, Albalak R, Grosse S, Repace J (2003). Secondhand smoke exposure and blood lead levels in U.S. children. Epidemiology.

[b25-ehp-117-468] Mielke HW (1999). Lead in the inner cities. Am Sci.

[b26-ehp-117-468] NCHS (National Center for Health Statistics) (2006a). National Health and Nutrition Examination Survey: NHANES 1999–2000.

[b27-ehp-117-468] NCHS (National Center for Health Statistics) (2006b). National Health and Nutrition Examination Survey: NHANES 2001–2002.

[b28-ehp-117-468] NCHS (National Center for Health Statistics) (2006c). National Health and Nutrition Examination Survey: NHANES 2003–2004.

[b29-ehp-117-468] Office of Management and Budget (1978). Statistical Policy Directive No. 14. Definition of Poverty for Statistical Purposes.

[b30-ehp-117-468] Pirkle JL, Brody DJ, Gunter EW, Kramer RA, Paschal DC, Flegal KM (1994). The decline in blood lead levels in the United States. The National Health and Nutrition Examination Surveys (NHANES). JAMA.

[b31-ehp-117-468] Rabinowitz M, Leviton A, Bellinger D (1985). Home refinishing, lead paint, and infant blood lead levels. Am J Public Health.

[b32-ehp-117-468] Raymond JS, Anderson R, Feingold M, Homa D, Brown MJ (2007). Risk for elevated blood lead levels in 3- and 4-year-old children. Matern Child Health J.

[b33-ehp-117-468] Reissman DB, Matte TD, Gurnitz KL, Kaufmann RB, Leighton J (2002). Is home renovation or repair a risk factor for exposure to lead among children residing in New York City?. J Urban Health.

[b34-ehp-117-468] Teekens R, Koerts J (1972). Some statistical implications of the log transformation of multiplicative models. Econometrica.

[b35-ehp-117-468] Tong SL, Baghurst PA, McMichael AJ, Sawyer MG, Mudge J (1996). Lifetime exposure to environmental lead and children’s intelligence at 11–13 years: the Port Pirie cohort study. BMJ.

[b36-ehp-117-468] U.S. EPA (1995). Report on the HUD National Survey of Lead-based Paint in Housing (Base Report).

[b37-ehp-117-468] U.S. EPA (1998). Risk Analysis to Support Standards for Lead in Paint, Dust, and Soil. Office of Pollution Prevention and Toxics.

[b38-ehp-117-468] U.S EPA (U.S. Environmental Protection Agency) (2001). Identification of Dangerous Levels of Lead. Final Rule. 40 CFR 745. Fed Reg.

[b39-ehp-117-468] U.S EPA (U.S. Environmental Protection Agency) (2008). Lead; Renovation, Repair and Painting. Final Rule. 40 CFR Part 745. Fed Reg.

[b40-ehp-117-468] Wasserman GA, Liu X, Lolacono NJ, Factor-Litvak P, Kline JK, Popovac D (1997). Lead exposure and intelligence in 7-year-old children: the Yugoslavia Prospective Study. Environ Health Perspect.

[b41-ehp-117-468] Wilson J, Dixon S, Galke W, McLaine P (2007). An investigation of dust lead sampling locations and children’s blood lead levels. J Expo Sci Environ Epidemiol.

[b42-ehp-117-468] Wilson J, Pivetz T, Ashley P, Jacobs D, Strauss W, Menkedick J (2006). Evaluation of HUD-funded lead hazard control treatments at 6 years post-intervention. Environ Res.

